# Atypical Histiocytoid Cells and Multinucleated Giant Cells in Fine-Needle Aspiration Cytology of the Thyroid Predict Lymph Node Metastasis of Papillary Thyroid Carcinoma

**DOI:** 10.3390/cancers11060816

**Published:** 2019-06-12

**Authors:** Ji Eun Choi, Ja Seong Bae, Dong-Jun Lim, So Lyung Jung, Chan Kwon Jung

**Affiliations:** 1 Department of Hospital Pathology, College of Medicine, The Catholic University of Korea, Seoul 06591, Korea; b612elf@gmail.com; 2 Department of Pathology, Design Hospital, Jeonju-si, Jeollabuk-do 54910, Korea; 3 Cancer Research Institute, College of Medicine, The Catholic University of Korea, Seoul 06591, Korea; drbae@catholic.ac.kr (J.S.B.); ldj6026@catholic.ac.kr (D.-J.L.); 4 Department of Surgery, College of Medicine, The Catholic University of Korea, Seoul 06591, Korea; 5 Division of Endocrinology and Metabolism, Department of Internal Medicine, College of Medicine, The Catholic University of Korea, Seoul 06591, Korea; 6 Department of Radiology, College of Medicine, The Catholic University of Korea, Seoul 06591, Korea; sljung1@catholic.ac.kr

**Keywords:** thyroid cytopathology, liquid-based preparation, fine needle aspiration, papillary carcinoma, lymph node metastasis

## Abstract

Preoperative detection of cervical lymph node metastasis in papillary thyroid carcinoma (PTC) is crucial for determining the surgical strategy to prevent locoregional recurrence of the disease. We identified the cytological predictors of lymph node metastasis in 222 consecutive patients with PTC using fine-needle aspiration cytology (FNAC) of the thyroid. Cervical lymph node metastases occurred in 99 (44.6%) of 222 PTC patients. Lymph node metastasis was significantly associated with tumor multifocality (*p* = 0.003), and high cellularity (*p* = 0.021), atypical histiocytoid cells (*p* < 0.001), and multinucleated giant cells (*p* < 0.001) in thyroid FNAC. The *BRAF* V600E mutation was marginally associated with lymph node metastasis (*p* = 0.054). Multivariate analysis revealed that atypical histiocytoid cells (odds ratio = 2.717; *p* = 0.001) and multinucleated giant cells (odds ratio = 3.070; *p* = 0.031) were independent predictors of lymph node metastasis in patients with PTC. In a subgroup analysis of 164 patients with microcarcinomas, atypical histiocytoid cells (odds ratio = 2.761; *p* = 0.005) was an independent predictor of lymph node metastasis. Cytological detection of atypical histiocytoid cells and multinucleated giant cells on thyroid FNAC can be used to preoperatively predict cervical lymph node metastasis in patients with PTC.

## 1. Introduction

Papillary thyroid cancer (PTC) represents 80–90% of all thyroid cancers [1]. Cervical lymph node metastasis occurs in 30% to 50% of patients with PTC and is related to local tumor recurrence after cancer surgery, and disease-specific mortality [1,2,3,4]. Preoperative detection of lymph node metastasis is crucial for surgical planning of PTC. Currently, ultrasonography is the best modality for the screening of thyroid nodules and cervical lymph nodes [1,2,5]. Ultrasound-guided fine-needle aspiration cytology (FNAC) has been considered the standard method for preoperative diagnosis of suspicious lymph nodes in PTC patients [1]. However, only 20–40% of patients with cervical lymph node metastasis can be preoperatively diagnosed using ultrasonography [6,7,8]. 

Previous studies reported that cervical lymph node metastasis correlates with the histopathologic characteristics of PTC including lymphatic invasion, psammoma bodies, micropapillary features, dyscohesive tumor cells, hobnail cells, and tall cells [9,10,11]. However, these histopathologic findings can be determined in surgical specimens postoperatively. Few studies have developed predictive models for lymph node metastasis using cytologic findings in preoperative thyroid FNAC specimens. Psammoma bodies are less frequently detected in cytologic preparations (4% to 20%), compared with histopathology (40–60%) [12,13]. We reported that the cytologic features of tall cells were easily detected in liquid-based cytology but tall cell features alone were not reliable in predicting lymph node metastasis of PTC [14]. 

Atypical histiocytoid cells found in thyroid FNAC are tumor cells with a histiocytoid or epithelioid appearance, and dark to vesicular nuclei and abundant cytoplasm [15,16]. Atypical histiocytoid cells were more frequently identified in metastatic lymph nodes than in primary PTC [15,17]. When cytologic features of atypical histiocytoid cells were correlated with histopathology, atypical histiocytoid cells were found in solid areas as well as in the cystic component of PTC [16]. Multinucleated giant cells are frequently found in PTC. A previous study conducted with resected specimens of PTC reported that multinucleated giant cells were associated with larger tumor size and extrathyroidal extension of PTC [18]. Little is known, however, about the significance of atypical histiocytoid cells and multinucleated giant cells observed in thyroid FNAC. 

We, therefore, evaluated the cytomorphologic features of PTC related to lymph node metastasis in preoperative thyroid FNAC specimens.

## 2. Results

### 2.1. Correlation between Clinicopathologic Features and Lymph Node Metastasis

The clinicopathological characteristics of the patient population are summarized in Table 1. Of the 222 patients with PTC, 165 (74.3%) were classic PTC, 21 (9.5%) classic PTC with tall cell features, 13 (5.9%) infiltrative follicular variants, and 11 (5.0%) tall cell variants. Cervical lymph node metastasis was found in 99 (44.6%) patients.

Cervical lymph node metastasis was not associated with age (*p* = 0.107) or sex (*p* = 0.923). Cervical lymph node metastasis was more frequently found in tumors larger than 1.0 cm (*p* = 0.012) and multifocal tumors (*p* = 0.003), as shown in Table 2. 

### 2.2. Correlation between Cytomorphologic Features and Lymph Node Metastasis

**Figure 1:**
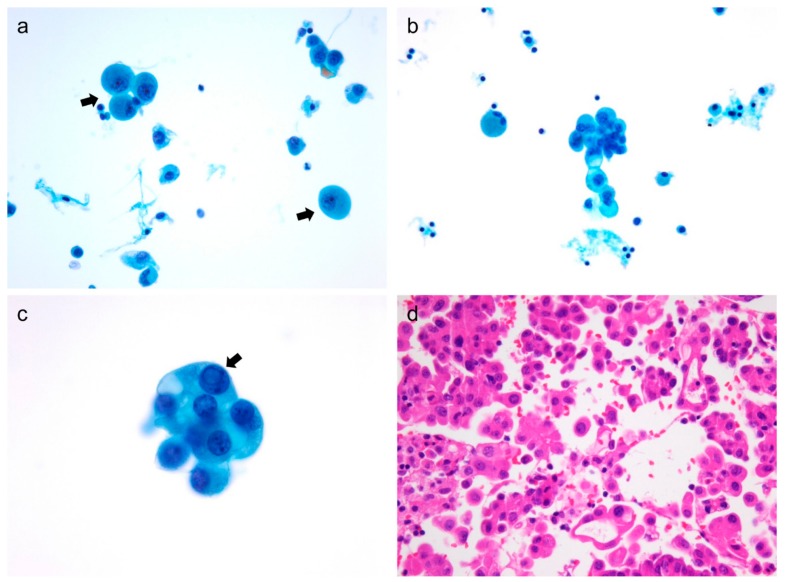
Cytologic features and corresponding histology of atypical histiocytoid tumor cells in papillary thyroid carcinoma (PTC). (**a**) Atypical histiocytoid tumor cells (arrows) contain abundant granular cytoplasm and lack the typical nuclear features of PTC (ThinPrep, Papanicolaou stain, ×400). (**b**) Atypical histiocytoid cells are clustered and show abundant and vacuolated cytoplasm (ThinPrep, Papanicolaou stain, ×400). (**c**) In a small cluster of atypical histiocytoid cells, a cell with abundant cytoplasm shows an intranuclear cytoplasmic pseudoinclusion (arrow) (ThinPrep, Papanicolaou stain, ×1000). (**d**) Corresponding histologic images reveal isolated or dyscohesive cells and micropapillary features (hematoxylin and eosin stain, ×400).

**Figure 2:**
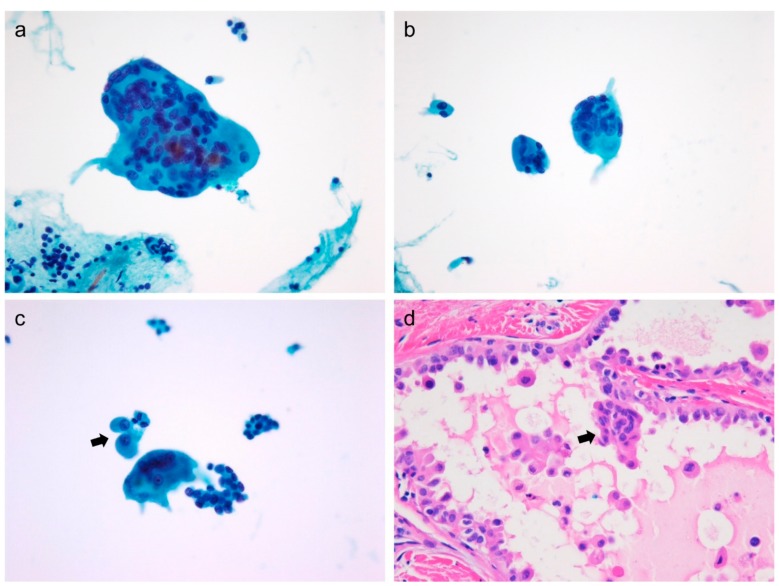
Cytologic features and corresponding histology of multinucleated giant cells in papillary thyroid carcinoma (PTC). (**a** and **b**) Multinucleated giant cells and tumor cells are seen in fine-needle aspiration of PTC. Multinucleated giant cells carry variable number of nuclei (ThinPrep, Papanicolaou stain, ×400). (**c**) Atypical histiocytoid cells (arrow) often accompany multinucleated giant cells (ThinPrep, Papanicolaou stain, ×400). (**d**) Corresponding histologic image shows a multinucleated giant cell (arrow) and dyscohesive tumor cells within the follicular space (hematoxylin and eosin stain, ×400).

Cytomorphogic findings such as cellularity, atypical histiocytoid cells (Figure 1), and multinucleated giant cells (Figure 2) observed in thyroid FNAC were significantly associated with cervical lymph node metastasis (*p* = 0.021, *p* < 0.001, and *p* < 0.001, respectively), as shown in Table 2. 

### 2.3. Correlation between BRAF Mutation and Lymph Node Metastasis

*BRAF* molecular testing was carried out in 203 (91.9%) of 222 patients. The *BRAF* V600E mutation was marginally associated with cervical lymph node metastasis (*p* = 0.054). 

### 2.4. Multivariate Analysis

In multivariate logistic regression analysis, atypical histiocytoid cells (odds ratio [OR], 2.717; *p* = 0.001), multinucleated giant cells (OR, 3.070; *p* = 0.031), and tumor multifocality (OR, 2.604; *p* = 0.002) were significant factors associated with cervical lymph node metastasis, as shown in Table 3. 

### 2.5. Cytomorphological Analysis of Atypical Histiocytoid Cells

Atypical histiocytoid cells in thyroid FNAC were more frequently detected in moderate-to-highly cellular aspirates (*p* < 0.001) and associated with moderate-to-high degree isolated tumor cells (*p* = 0.002), tall cells (*p* < 0.001), and multinucleated giant cells (*p* < 0.001), as shown in Table 4. However, the presence of atypical histiocytoid cells was not associated with macrophages, psammoma bodies, and *BRAF* V600E mutations.

### 2.6. Sub-Analysis of Patients with Microcarcinoma

We further analyzed the cytomorphologic features associated with lymph node metastasis in 164 patients with sub-centimeter PTC. Cervical lymph node metastasis and lateral lymph node metastasis was found in 65 (39.6%) and 15 (9.1%) patients, respectively. Cervical lymph node metastasis in patients with microcarcinoma was significantly associated with the presence of atypical histiocytoid cells (*p* = 0.005), abundant multinucleated giant cells (*p* = 0.027), and *BRAF* V600E mutation (*p* = 0.041), as shown in Table 5. 

In multivariate logistic regression analysis, atypical histiocytoid cells (OR, 2.761; *p* = 0.005) was significant factor associated with cervical lymph node metastasis, as shown in Table 6. 

## 3. Discussion

Cervical lymph node metastasis frequently occurs in patients with PTC and is the main cause of disease recurrence [4,19,20]. Because surgical management of PTC has been further minimized and individualized, preoperative evaluation of cervical lymph node metastasis is important in determination of the extent of surgical resection [21,22]. In this study, we found that the presence of atypical histiocytoid cells and multinucleated giant cells in thyroid FNAC is a predictive marker for cervical lymph node metastasis. 

In previous studies, atypical histiocytoid cells were frequently found in aspirates obtained from metastatic lymph nodes. Canepa et al. reported that atypical histiocytoid cells were identified in 68% of FNACs involving cervical lymph node metastases from PTC [17]. The atypical histiocytoid tumor cells are predominantly found in cystic metastases of PTC to lymph nodes and represent a potential diagnostic pitfall leading to a false-negative diagnosis due to the lack of typical nuclear features of PTC [17]. However, there is little information on their diagnostic and predictive value in preoperative thyroid FNAC specimens. In our study, we identified atypical histiocytoid cells in 122 (55.0%) of 222 aspirates in primary PTC and found a significant correlation between the presence of atypical histiocytoid cells in thyroid FNAC and cervical lymph node metastasis after surgical resection.

The presence of abundant macrophages is one of the first clues suggesting cystic changes of thyroid nodule. The FNAC of the cystic variant of PTC is characterized by watery fluid, thin colloids, abundant macrophages, and hypervacuolated (histiocytoid) tumor cells [13]. However, atypical histiocytoid cells can be seen in FNAC of PTC, even when cystic degeneration is absent [16]. We found no correlation between atypical histiocytoid cells and macrophages in the aspirates of primary PTC while atypical histiocytoid cells were associated with cellularity, isolated tumor cells, and tall cells. When we further correlated cytological findings with corresponding histology, atypical histiocytoid cells were frequently found in PTC cases with abundant dyscohesive cells and micropapillary features, as shown in Figure 1. Previous studies reported that dyscohesive cells and micropapillary features in the surgical specimens of PTC were correlated with lymph node metastasis [10,11]. Therefore, we suggest that atypical histiocytoid cells in FNAC of primary PTC do not merely represent cystic variants of PTC but serve as potential biomarkers for the prediction of cervical lymph node metastasis in patients with PTC.

Multinucleated giant cells derived from histiocytic lineage are seen in benign and malignant thyroid nodules [13,18,23]. In previous studies, multinucleated giant cells in PTC were found twice as frequently as benign nodules [23]. Their nuclei were characterized by varying size, shape, and number. PTC patients with an abundance of multinucleated giant cells manifested frequent extrathyroidal extensions and larger tumor size compared with those with few or no multinucleated giant cells [18]. Multinucleated giant cells are seldom seen in FNAC of non-invasive follicular thyroid neoplasm with papillary like nuclear features [24]. Multinucleated giant cells identified in FNAC were consistent with giant cells found in corresponding histologic specimens, as shown in Figure 2. These results show the prognostic value of multinucleated giant cells. We found that the presence of moderate-to-high number of giant cells in FNAC was associated with lymph node metastasis and the presence of atypical histiocytoid cells. 

Psammoma bodies, characteristic features of PTC, are less frequently seen in liquid-based preparations than in conventional smears and histologic specimens [25]. In our study, psammoma bodies were found in only 11 (5.0%) of 222 liquid-based cytology specimens. Tall cells are easily identified in liquid-based preparations and their presence in FNAC was strongly correlated with tall cell variants of PTC [14,26]. However, there was no association between tall cells and lymph node metastasis in our present and previous studies [18].

Low cellularity in FNAC may be attributed to thyroid tumor with hypervascularture, tumor fibrosis, or cystic degenerative changes [27]. Low cellularity hinders accurate interpretation of cytomorphologic findings. Cytologic features associated with malignancy may also be absent in the specimens with low cellularity. We observed that atypical histiocytoid cells and multinucleated giant cells were less frequent in tumors with low cellularity than in specimens with moderate-to-high cellularity. Cervical lymph node metastasis was less frequently detected in patients with low cellularity in FNAC. Therefore, cellularity per se may predict lymph node metastasis of PTC. However, further studies are needed to evaluate the reasons underlying the low cellularity and its association with lymph node metastasis.

Prophylactic central lymph node dissection for clinically node-negative (cN0) PTC was recommended in the previous 2016 American Thyroid Association (ATA) guidelines [28], but not recommended in the 2009 and 2015 revised ATA guidelines [1,22]. The main arguments against prophylactic central lymph node dissection for PTC are the lack of survival benefit and the risk of complications [29]. A recent meta-analysis revealed that the pooled rate of central lymph node metastasis of PTC was 48% (958/1996) by patient-based analysis of nine studies [30]. The pooled sensitivity and specificity of ultrasound for the detection of metastatic central lymph node were 33% (95% confidence interval: 31–35%) and 93% (95% confidence interval: 92–94%), respectively [30]. The meta-analysis study suggested that prophylactic central lymph node metastasis for PTC should be performed, considering the reported high incidence of central lymph node metastasis and the poor performance of preoperative ultrasound in detecting central lymph node metastasis. However, the effect of prophylactic central lymph node dissection on recurrence or survival rates remains uncertain and requires further studies with long-term follow-up. In the present study, cervical lymph node metastases of PTC were found in 44.6% of 222 consecutive patients with routine prophylactic central lymph node dissection, which falls within the range of previous studies reporting that cervical lymph node metastasis occurs in 20% to 50% of patients with differentiated thyroid cancer [1]. There were no major postoperative complications such as permanent hypoparathyroidism, dysphonia and hemorrhage. The sensitivity and specificity of cytomorphologic features of primary PTC for predicting cervical lymph node metastasis were 69.9% and 56.9% for atypical histiocytoid cells and 21.2% and 95.1% for multinucleated giant cells, respectively. Our results showed that atypical histiocytod cells had a higher sensitivity and multinucleated giant cell had a higher specificity in the detection of cervical lymph node metastasis of PTC than previously published diagnostic performance of ultrasound. Further studies are warranted to prove whether the combination of cytomorphologic features and ultrasound imaging predicts cervical lymph node metastasis of PTC more accurately.

The association of *BRAF* V600E mutation and lymph node metastasis in PTC remains controversial. Clinicopathological factors that may affect the *BRAF* mutation rate in PTC are age, sex, histologic variants, tumor size, extrathyroidal extension, multifocality, and Hashimoto thyroiditis [31,32]. Other factors that may affect the mutation rate results are the method of molecular analysis and type of sample to be tested [32]. Sanger sequencing is considered a gold standard for mutation analysis, but its sensitivity to detect mutation is 15% to 20% mutant allele frequency [33]. The sensitivity of next generation sequencing (NGS) is higher (down to 1% mutant allele frequency) than that of Sanger sequencing, and NGS can simultaneously test multiple genes by using low amount of input nucleic acids [33,34]. Therefore, targeted NGS test is being increasingly used in molecular diagnosis of thyroid FNAC samples [32,34]. In the present study, *BRAF* mutational status was analyzed by Sanger sequencing using DNA extracted from tissue sections corresponding to the FNAC from PTC nodules. The *BRAF* V600E mutation was detected in 156 (76.8%) of 203 tumors and was marginally associated with cervical lymph node metastasis (*p* = 0.054). Although many studies have shown an association between *BRAF* V600E mutation and lymph node metastasis in PTC, the 2015 ATA guidelines note that, “the presence of *BRAF* V600E mutation in the primary PTC should not impact the decision for prophylactic central neck dissection” due to low positive predictive value of the test for disease recurrence [1]. Therefore, further studies are needed to establish the clinical utility of the *BRAF* V600E mutation as a predictive biomarker for lymph node metastasis in PTC.

## 4. Materials and Methods

### 4.1. Patients and Clinical Samples

This study evaluated patients who underwent thyroid FNAC and thyroidectomy at Seoul St. Mary’s Hospital from January 2012 to December 2012. Prophylactic central lymph node dissection for PTC was routinely performed by experienced endocrine surgeons. A total of 222 consecutive patients were enrolled according to the following criteria: (1) liquid-based cytology slides with sufficient cellular material for cytologic evaluation; (2) pathologic diagnosis of PTC; and (3) prophylactic or therapeutic lymph node dissection. Patient age ranged from 24 to 79 years (mean age of 49.5 years) at the time of diagnosis. Tumor size ranged from 0.3 cm to 3.5 cm (mean of 0.9 cm). Of the 222 patients with PTC, 164 (73.9%) showed micro PTC (≤1.0 cm in size). This study was approved by the Institutional Review Board of Seoul St. Mary’s Hospital of the Catholic University of Korea (KC16SISI0104).

### 4.2. Cytomorphologic Evaluation

All cases of FNAC of thyroid nodules were performed by radiologists using 23-gauge needles under real-time ultrasound guidance. FNAC materials were processed using ThinPrep method (Hologic Inc., Marlborough, MA, USA) or SurePath method (BD Diagnostics, Franklin Lakes, NJ, USA) and stained with Papanicolaou stain. 

All thyroid FNAC slides were examined independently by two experienced pathologists (Ji Eun Choi and Chan Kwon Jung) who were blinded to the clinical, pathologic, and molecular findings. Cellularity was estimated as low, moderate, or high. The following cytologic features were graded as absent, low (1–2 per slide), moderate (3–5 per slide), or high (≥5 per slide) in number: isolated tumor cells, atypical histiocytoid cells, tall cells, psammoma bodies, multinucleated giant cells, and macrophages. Atypical histiocytoid cells were defined as tumor cells with enlarged round nuclei, abundant cytoplasm, occasional nucleoli and intranuclear pseudoinclusions, and lack of nuclear grooves (Figure 1). Tall cells included tumor cells manifesting nuclear features of PTC with a height at least twice their width. Discrepancy in the observations of the 2 reviewers was resolved based on consensus.

### 4.3. BRAF V600E Mutation Analysis

*BRAF* mutation analysis was performed using formalin-fixed paraffin embedded tissue sections corresponding to the FNAC from PTC nodules using Sanger sequencing. Exon 15 of the *BRAF* gene was amplified via PCR using specific primers (forward 5′-TCATAATGCTTGCTCTGATAGGA-3′; reverse 5′-GGCCAAAAATTTAATCAGTGGA-3′) as previously described [35,36]. Sanger sequencing of PCR amplicons was performed using the same PCR primers.

### 4.4. Statistical Analysis

To analyze the correlation between clinical and thyroid cytomorphologic features, and lymph node metastasis, χ^2^-test or Fisher’s exact test was used when appropriate. The Student’s *t*-test was used to compare the continuous variables. To identify factors that independently predicted the risk of lymph node metastasis, multivariate logistic regression analysis was performed with backward selection methods to avoid overfitting. *p*-value < 0.05 indicated statistical significance. Statistical analysis was performed with SPSS software (IBM SPSS Statistics for Windows, Version 22.0., IBM Corp., Armonk, NY, USA).

## 5. Conclusions

Atypical histiocytoid cells and multinucleated giant cells can be readily identified in liquid-based preparations of PTC. These cytomorphologic findings of FNAC can be used to predict cervical lymph node metastasis in PTC patients.

## Figures and Tables

**Table 1:** Demographic and clinicopathological characteristics of 222 patients with papillary thyroid carcinoma.

Characteristics	No. of Cases
Age (year, mean ± standard deviation)	49.5 ± 12.8
Sex	
Female	172 (77.5%)
Male	50 (22.5%)
Tumor size	
≤1 cm	164 (73.9%)
>1 cm	58 (26.1%)
Histologic subtype	
Classic papillary	165 (74.3%)
Classic papillary with tall cell features	21 (9.5%)
Infiltrative follicular	13 (5.9%)
Tall cell	11 (5.0%)
Invasive encapsulated follicular	8 (3.6%)
Oncocytic	2 (0.9%)
Cribriform morular	1 (0.5%)
Warthin-like	1 (0.5%)
Multifocality	
Unifocal	112 (50.5%)
Multifocal	110 (49.5%)
Extrathyroidal extension	
Absent	133 (59.9%)
Microscopic	82 (36.9%)
Gross	7 (3.2%)
pN stage	
pN0	123 (55.4%)
pN1a	74 (33.3%)
pN1b	25 (11.3%)
*BRAF* V600E mutation ^1^	
Absent	47/203 (23.2%)
Present	156/203 (76.8%)

^1^*BRAF* mutation testing was available for 203 patients.

**Table 2:** Correlation between clinicopathological and cytomorphologic features and lymph node metastasis in 222 patients with papillary thyroid carcinoma.

Variable	Cervical Lymph Node Metastasis		
	Absent (*n* = 123)	Present (*n* = 99)	*p*-Value
Age (year, mean ± standard deviation)	50.8 ± 12.6	48.0 ± 12.9	0.107
Sex			0.923
Female	95 (77.2%)	77 (77.8%)	
Male	28 (22.8%)	22 (22.2%)	
Tumor size			0.012
≤1 cm	99 (80.5%)	65 (65.7%)	
>1 cm	24 (19.5%)	34 (34.3%)	
Multifocality			0.003
Unifocal	73 (59.3%)	39 (39.4%)	
Multifocal	50 (40.7%)	60 (60.6%)	
Cellularity			0.021
Low	33 (26.8%)	14 (14.1%)	
Moderate/high	90 (73.2%)	85 (85.9%)	
Isolated tumor cells			0.657
Absent/low	83 (67.5%)	64 (64.6%)	
Moderate/high	40 (32.5%)	35 (35.4%)	
Atypical histiocytoid cells			<0.001
Absent	70 (56.9%)	30 (30.3%)	
Present	53 (43.1%)	69 (69.7%)	
Tall cells			0.952
Absent/low	104 (84.6%)	84 (84.8%)	
Moderate/high	19 (15.4%)	15 (15.2%)	
Multinucleated giant cells			<0.001
Absent/low	117 (95.1%)	78 (78.8%)	
Moderate/high	6 (4.9%)	21 (21.2%)	
Macrophages			0.786
Absent/low	104 (86.4%)	85 (85.9%)	
Moderate/high	19 (15.4%)	14 (14.1%)	
Psammoma bodies			0.224
Absent	119 (96.7%)	92 (92.9%)	
Present	4 (3.3%)	7 (7.1%)	
*BRAF* V600E mutation ^1^			0.054
Wild	31/109 (28.4%)	16/94 (17.0%)	
Mutant	78/109 (71.6%)	78/94 (83.0%)	

^1^*BRAF* mutation testing was available for 203 patients.

**Table 3:** Multivariate logistic regression analyses for prediction of cervical lymph node metastasis in papillary thyroid carcinoma.

Characteristic	Odds Ratio	95% Confidence Interval	*p*-Value
Tumor size ≤1 cm vs. >1 cm	1.425	0.712–2.853	0.318
Multifocality Unifocal vs. multifocal	2.604	1.441–4.705	0.002
Cellularity Low vs. moderate/high	1.446	0.680–3.077	0.338
Atypical histiocytoid cells Absent vs. present	2.717	1.468–5.029	0.001
Multinucleated giant cells Absent/low vs. moderate/high	3.070	1.109–8.497	0.031

**Table 4:** Analysis of cytomorphologic features associated with atypical histiocytoid cells in the aspirates of papillary thyroid carcinoma.

Variable	Atypical Histiocytoid Cells		
	Absent (*n* = 100)	Present (*n* = 122)	*p*-Value
Cellularity			<0.001
Low	32 (32.0%)	15 (12.3%)	
Moderate/high	68 (68.0%)	107 (87.7%)	
Isolated tumor cells			0.002
Absent/low	77 (77.0%)	70 (57.4%)	
Moderate/high	23 (23.0%)	52 (42.6%)	
Tall cells			<0.001
Absent/low	94 (94.0%)	94 (77.0%)	
Moderate/high	6 (6.0%)	28 (23.0%)	
Multinucleated giant cells			<0.001
Absent/low	97 (97.0%)	98 (80.3%)	
Moderate/high	3 (3.0%)	24 (19.7%)	
Macrophages			0.277
Absent/low	88 (88.0%)	101 (82.8%)	
Moderate/high	12 (12.0%)	21 (17.2%)	
Psammoma bodies			0.352
Absent	97 (97.0%)	114 (93.4%)	
Present	3 (3.0%)	8 (6.6%)	
*BRAF* V600E mutation ^1^			0.482
Wild	22/86 (25.6%)	25/117 (21.4%)	
Mutant	64/86 (74.4%)	92/117 (78.6%)	

^1^*BRAF* mutation testing was available for 203 patients.

**Table 5:** Correlation between clinicopathological and cytomorphologic features and lymph node metastasis in 164 patients with papillary thyroid microcarcinoma.

Variable	No. of Cases	Cervical Lymph Node Metastasis		
		Absent (*n* = 99)	Present (*n* = 65)	*p*-Value
Age (year, mean ± standard deviation)	49.8 ± 12.5	50.7 ± 12.1	48.4 ± 13.0	0.255
Sex				0.525
Male	37 (22.6%)	24 (24.2%)	13 (20.0%)	
Female	127 (77.4%)	75 (75.8%)	52 (80.0%)	
Multifocality				0.080
Unifocal	87 (53.0%)	58 (58.6%)	29 (44.6%)	
Multifocal	77 (47.0%)	41 (41.4%)	36 (55.4%)	
Cellularity				0.269
Low	43 (26.2%)	29 (29.3%)	14 (21.5%)	
Moderate/high	121 (73.8%)	70 (70.7%)	51 (78.5%)	
Isolated tumor cells				0.682
Absent/low	114 (69.5%)	70 (70.7%)	44 (67.7%)	
Moderate/high	50 (30.5%)	29 (29.3%)	21 (32.3%)	
Atypical histiocytoid cells				0.005
Absent	80 (48.8%)	57 (57.6%)	23 (35.4%)	
Present	84 (51.2%)	42 (42.4%)	42 (64.6%)	
Tall cells				0.628
Absent/low	139 (84.8%)	85 (85.9%)	54 (83.1%)	
Moderate/high	25 (15.2%)	14 (14.1%)	11 (16.9%)	
Multinucleated giant cells				0.027
Absent/low	153 (93.3%)	96 (97.0%)	57 (87.7%)	
Moderate/high	11 (6.7%)	3 (3.0%)	8 (12.3%)	
Macrophages				0.628
Absent/low	139 (84.8%)	85 (85.9%)	54 (83.1%)	
Moderate/high	25 (15.2%)	14 (14.1%)	11 (16.9%)	
Psammoma bodies				0.485
Absent	155 (94.5%)	95 (96.0%)	60 (92.3%)	
Present	9 (5.5%)	4 (4.0%)	5 (7.7%)	
*BRAF* V600E mutation ^1^				0.041
Wild	34 (22.8%)	25 (28.7%)	9 (14.5%)	
Mutant	115 (77.2%)	62 (71.3%)	53 (85.5%)	

^1^*BRAF* mutation testing was available for 149 patients.

**Table 6:** Multivariate logistic regression analyses for prediction of cervical lymph node metastasis in papillary thyroid microcarcinoma.

Characteristics	Odds Ratio	95% Confidence Interval	*p*-Value
Multifocality Unifocal vs. multifocal	2.415	1.205–4.842	0.013
Cellularity Low vs. moderate/high	1.134	0.517–2.487	0.754
Atypical histiocytoid cells Absent vs. present	2.761	1.350–5.648	0.005
Multinucleated giant cells Absent/low vs. moderate/high	3.703	0.908–15.106	0.068
